# Sex-dependent effects of ultra-low-dose-THC preventive treatment on neuroinflammation and cognitive decline in 5xFAD mice

**DOI:** 10.1186/s13293-025-00815-3

**Published:** 2026-01-03

**Authors:** Keren Nitzan, Ziv Bentulila, Noa Bregman-Yemini, Niv Ayalon, Dekel David, Emanuela Break, Yossi Sarne, Ravid Doron

**Affiliations:** 1 Department of Education and Psychology, The Open University, 1 University Road, Ra’anana, Israel; 2 Department of Psychology, The Hebrew University, Jerusalem, Israel; 3 Department of Physiology and Pharmacology, Sackler Faculty of Medicine, Tel Aviv University, Tel Aviv, Israel

**Keywords:** Alzheimer, Neuroinflammation, Sex-differences, Ultra-low-dose tetrahydrocannabinol

## Abstract

**Background:**

Alzheimer’s disease (AD) remains the most prevalent cause of dementia, yet no existing treatment effectively prevents its onset. Current therapies primarily aim to slow disease progression or manage symptoms, leaving a critical gap in preventive strategies. Recent findings suggest that ultra-low-dose tetrahydrocannabinol (ULD-THC) may exert neuroprotective effects without the adverse consequences associated with chronic THC use. This study investigates whether preventive ULD-THC treatment can mitigate neuroinflammation and early cognitive decline in the 5xFAD mouse model of AD, with a specific focus on sex differences in treatment response.

**Methods:**

Male and female 5xFAD mice received monthly ULD-THC injections from 3 to 5 months of age, before significant pathology emerged. At 6 months, behavioral assessments were conducted, followed by molecular analyses of hippocampal and prefrontal cortex (PFC) tissue.

**Results:**

Results indicated that ULD-THC attuned AD-related cognitive decline in both males and females, with sex-specific neuroinflammatory responses. Males exhibited reduced hippocampal inflammation, whereas females showed reduced inflammation in the PFC, suggesting distinct neuroprotective mechanisms across sexes.

**Conclusions:**

These findings highlight ULD-THC’s potential as a preventive strategy for AD, emphasizing the importance of sex-dependent therapeutic approaches. By attenuating neuroinflammatory processes before cognitive deficits fully manifest, ULD-THC offers a novel, biologically targeted approach to AD prevention. Future research should explore its long-term efficacy and translational potential in clinical settings.

**Supplementary Information:**

The online version contains supplementary material available at 10.1186/s13293-025-00815-3.

## Highlights

- **ULD-THC modulates neuroinflammationand glial activity**: Treatment reduced astrocytic (GFAP) and microglial (AIF1) activation, alongside changes in additional inflammation-related markers, demonstrating a targeted biological response in key AD-affected brain regions.
- **Sex-specific neuroinflammatory effects**: Males exhibited reduced hippocampal inflammation, while females showed a decrease in PFC inflammation, indicating distinct region-dependent responses.
- **Preventive potential of ULD-THC**: Early administration of ULD-THC, before significant pathology emerged, successfully reduced AD-related neuroinflammation.

**Supplementary Information:**

The online version contains supplementary material available at 10.1186/s13293-025-00815-3.

## Main text

Alzheimer’s disease (AD) is the most common cause of dementia, with women being twice as likely to be affected compared to men. AD patients suffer from memory loss, poor judgment, confusion and neuropsychiatric disorders, accompanied by the accumulation of beta-amyloid (Aβ) and tau tangles in the brain [1]. Many changes have been identified in the AD brain, such as mitochondrial dysfunction [2], modification in the neurotrophic system [3], neuronal loss [4] and most notably – neuroinflammation [5, 6].

Although the mechanism of AD has been investigated for more than 50 years, currently, there are only a few available drugs for AD that can slow Aβ accumulation and treat some of the cognitive and emotional symptoms. In the past two to three years, new drugs have been approved for Alzheimer’s disease, including symptomatic treatments like Benzgalantamine (a cholinesterase inhibitor, FDA-approved in July 2024) and disease-modifying therapies like Lecanemab and Donanemab (anti-amyloid monoclonal antibodies, FDA-approved in July 2023 and July 2024, respectively) [7]. While these therapies mark significant progress, they do not prevent the disease. The distinction between slowing progression and true prevention is crucial, as no existing treatment can halt the pathological processes before they begin. Thus, while there have been advances in treating AD, there is still no way to prevent the disease [8].

In the last decade, studies have found many changes in the endocannabinoid system in AD [9–11], yielding several clinical [12–14] and pre-clinical [15–17] studies on the benefits of cannabis and its main compound − 9Δ-tetrahydrocannabinol (THC). However, the use of THC in patients is hindered by the harmful effects of high doses of chronic THC use [18]. One strategy that has been suggested to tackle this issue is to use an ultra-low dose of THC (ULD-THC) [19–21]. Indeed, we have recently shown that a single dose of ULD-THC has a beneficial effect on age-related cognitive decline in old female mice [16] and alleviated AD-related cognitive impairments in the 5xFAD AD mice model [17]. However, the potential of cannabinoids in the prevention of AD is still unexplored. The primary objective of this study was to evaluate the effects of preventive treatment of ULD-THC on neuroinflammation and cognitive deficits in male and female 5xFAD AD mice.

One of the known effects of THC treatment is the modulation of the immune system and reduction of neuroinflammation [22]. This can be important in AD, as the accumulation of Aβ exacerbates neuroinflammation in the AD brain [23], raising the levels of pro-inflammatory cytokines [24]. This chronic neuroinflammation causes a significant activation of microglial cells and astrocytes in the brain [25]. Microglia are the brain’s immune cells, and in response to neuronal injury or infection, microglia become activated and produce pro and anti-inflammatory cytokines. While microglia initially aim to clear Aβ, their sustained activation in AD accelerates disease progression [26]. Indeed, 4-month-old 5xFAD mice significantly increased hippocampal microglial expression and activation [27]. Astrocytes also play a crucial role in maintaining neuronal function and maturation, and can exhibit either neuroprotective or neurotoxic effects. Reactive astrocytes are a characteristic feature in AD patients’ brains and are also present in AD mouse model brains [28]. Interestingly, the activation of glial cells in the 5xFad model is different between males and females [29]. In addition to glial cells, several other immune-modulating proteins have been connected to AD neuroinflammation - Serum- and glucocorticoid-inducible kinase 1 (SGK1) and Tissue Inhibitor of Metalloproteinases 3 (TIMP-3), both of which play a complex part in glial-induced inflammation and are known to be upregulated in AD pathology. However, the effect of this upregulation in AD is not yet clear, and can be either pro-inflammatory or neuroprotective [30–32].

The 5xFAD model is a widely used AD mouse model, characterized by early Aβ accumulation and extensive brain pathology from 2 to 3 months of age, with cognitive decline becoming evident around six months [33]. The hippocampus and prefrontal cortex (PFC) are two key regions affected in AD [34, 35]. Interestingly, Female 5xFAD mice exhibit earlier PFC degradation progressing to hippocampus-related deficits in later stages [36]. By contrast, male mice show damage mainly in the hippocampus [36], 5xFad female mice are also prone to higher levels of inflammation compared to male 5xFad mice [37]. These sex-specific differences point to distinct neural strategies for memory and learning, influenced by both sex-dependent brain pathology and task demands [38].

Building on prior findings of memory improvement following ULD-THC treatment after disease onset [17], this study employs a preventive regimen to explore sex-specific molecular and behavioral responses to ULD-THC administered at earlier stages of AD pathology. We investigate here the preventive effects of ULD-THC on neuroinflammation and cognitive deficits in 5xFAD male and female mice and explore the molecular changes in both the hippocampus and PFC. 5xFAD mice start to exhibit early brain pathologies between 1.5 and 3 months, with behavioural phenotype staring at 6 months [39, 40]. Give our previous experiment indicate a beneficial effect of ULD-THC for up to 3–4 weeks after the single treatment, we employed a monthly ULD-THC treatments from 2 to 5 months of age were followed by behavioral assessments and molecular analyses at 6 months.

## Materials and methods

### Animals

80 male and female 5xFAD mouse model (Swedish K670N, M671L, Florida I716V, London V717I, and two mutations in the human presenilin-1 gene: M146L and L286V; stock number 34840), were kept in the vivarium at the Ein Kerem Hadassah Medical Center’s Psychobiology Lab of the Open University on a reverse 12 h light/dark cycle and provided with food and water ad libitum.

5xFAD male mice were bred with C57B/Rcc females, and offspring were genotyped by PCR analysis of tail DNA to identify the tg-mice. Non-Tg littermates were used as control WT mice. All experiments were performed during the dark phase (7:00–19:00) under red light. All experimental protocols were examined and approved by the Institutional Animal Care and Use Committee.

### Study design

To determine the prevention efficacy of ULD-THC in the 5xFAD mice model, 80 3-month-old male and female 5xFAD mice and their WT littermates were treated with three i.p. injections of either ULD-THC or vehicle (10 per group) once a month for 3 months from the age of 3 to 5 months. At the age of 6 months, 3 weeks after the last treatment, mice were tested for both short-term and long-term spatial memory. The mice underwent one test per day for 2 weeks, after which they were sacrificed for biochemical examination. All male and female mice were subjected to the same behavioral and biochemical tests following an identical timeline.

### Treatments

Δ9-THC (donated by Prof. Mechoulam, the Hebrew University, Jerusalem and by NIDA, USA) was dissolved from a stock solution in ethanol into a vehicle solution consisting of 1:1:18 ethanol: cremophor (Sigma-Aldrich): saline and was administered i.p at a dose of 0.002 mg/kg.

Mice were treated with a monthly i.p. injection of either ULD-THC or vehicle, starting before disease onset at the age of 3–5 months. Thus, each mouse was treated 3 times – once at the age of 3 months, once at the age of 4 months and once at the age of 5 months. At the age of 6 months, 3 weeks after the last treatment, the animals were assessed for cognitive deficits.

All experimenters were blinded to treatment during treatment and data collection.

### Behavioural tests

Mice were tested for two weeks in the following order: in the first week, the mice were tested first with the Open field habituation (OFH), then PLT (Place recognition test) and lastly Y-MAZE. In the second week, mice were tested in the Morris water maze (MWM.)

#### Morris water maze (MWM)

Learning ability and spatial memory were evaluated using MWM. This classical spatial learning assay was performed as previously described [17]. Briefly, the mouse was introduced into a round pool at different starting points, spatial cues (such as a black cross on the white wall, etc.) were visible for the mice, and was allowed 60 s to find the platform. The water was kept at a temperature of 23 degrees Celsius. Each mouse swam four times per day for three consecutive days. The time and route required for the mouse to find the immersed platform was recorded. On day 4 of the assay, the platform was removed, and the mouse was allowed to swim for 60 s (“probe test”). The time the mice spent in the platform zone (the location of the missing platform) was recorded.

#### Y maze (YM)

This test evaluated short-term working memory (PFC-dependent task) and was performed as previously described [2, 41]. Briefly, the Y-maze is a three-arm maze with all arms at equal angles, 30 cm in length and 5 cm in width with walls 12 cm high. Mice were initially placed in the middle, and the sequence of arm entries was recorded for each mouse over an 8-min period. The triads with all three arms represented (i.e., ABC, CAB, or BCA but not ABB) – were considered ‘correct triads’.

**Place recognition test (PLT):** This assay tests long-term memory and utilizes the natural tendency of mice to explore novel stimuli. It was performed as previously described [17]. Animals were placed in a familiar 40 cm × 40 cm × 40 cm open field box, with two objects (6*5*7 cm cube. A picture of the arena and the objects can be seen in the supplementary file in Fig. 4S). Briefly, the test comprises two parts: a familiarization session and a test session. During the familiarization session, the mouse was left to explore two identical objects for 5 min. 24 h later, the mouse was introduced into the arena for the test session, in which one of the familiar objects was placed in a new location (“place recognition”). The time spent exploring each object was recorded for 5 min.

**Open-field habituation (OFH):** This test evaluates long-term non-associative, non-aversive spatial learning by measuring the decrease in the exploratory activity of the animal in a test session carried out 24 h after the first exploration session (delta of 2nd session – 1 st session) [2]. In Brief, animals were exposed to a novel environment by placing them in a 40 cm × 40 cm × 40 cm open field box. The distance walked was measured for a 5-min period. Twenty-four hours later animals were re-exposed to the same environment and the distance they walked was recorded again. Bigger delta between days (shorter distance on the test session compared to the first session) represented intact learning.

The arenas of the Y-maze, OFH and PLT were cleaned with Alcohol between mice, in order to eliminate olfactory cues. All the behavioral assays were photographed using a video camera, and data were recorded and analyzed using the 13th Noldus EthoVision software (tracking the center-mass in MWM, Y-maze and OFH, and nose-point for place recognition test to verify only instances that the mouse was interested in the object were counted).

**RT-PCR:** After the last behavioral test, mice were euthanized using cervical dislocation, and the hippocampus and PFC tissue were removed and kept at −80c. Total RNA was extracted from the hippocampus using the PureLink RNA Mini Kit (Rhenium, Israel) according to the manufacturer’s instructions. SYBR Green real-time PCR primers was purchased from Agentek Israel. RT-PCR was performed with primers specific for tissue inhibitor of metalloproteinase 3 (Timp-3), Sgk1, allograft inflammatory factor 1 (Aif1) and glial fibrillary acidic protein (Gfap) (Agantek, Israel), and were assayed for expression using the Magnetic Induction Cycler (Mic) PCR Machine (biomolecularsystems). Absolute quantification was performed by comparing Ct values of the target genes to a standard curve generated using serial dilutions of known template concentrations.

**Data analysis:** All results are presented as mean ± standard error of the mean. All data were first checked for normality and equality of variance to ensure the assumptions of parametric tests were met. A two-sided paired Student’s t-test was used to examine the difference between the days/objects in the OFH and PLT. Repeated ANOVA was used for days 1–3 of the MWM, and one-way ANOVA for the Y-maze and all biological results. When applicable, results were further analyzed using LSD post-hoc analysis after significant ANOVA results. Extreme values (+/- 2 standard deviations) were excluded from the statistical analysis. Statistical analysis was performed using PRISM. The level of significance was set at *p* < 0.05.

## Results

### Preventive treatment with ULD-THC improves Spatial hippocampal-dependent memory in male 5xFAD mice

Mice received three monthly injections of ULD-THC (0.002 mg/kg) or vehicle before the disease onset, between the ages of 3–5 months. Three weeks after the last injection, mice were tested for short-term working memory (Y-Maze), long-term spatial memory (Novel location test and Morris water maze), and long-term associative learning (Open Field Habituation).

**Fig. 1:**
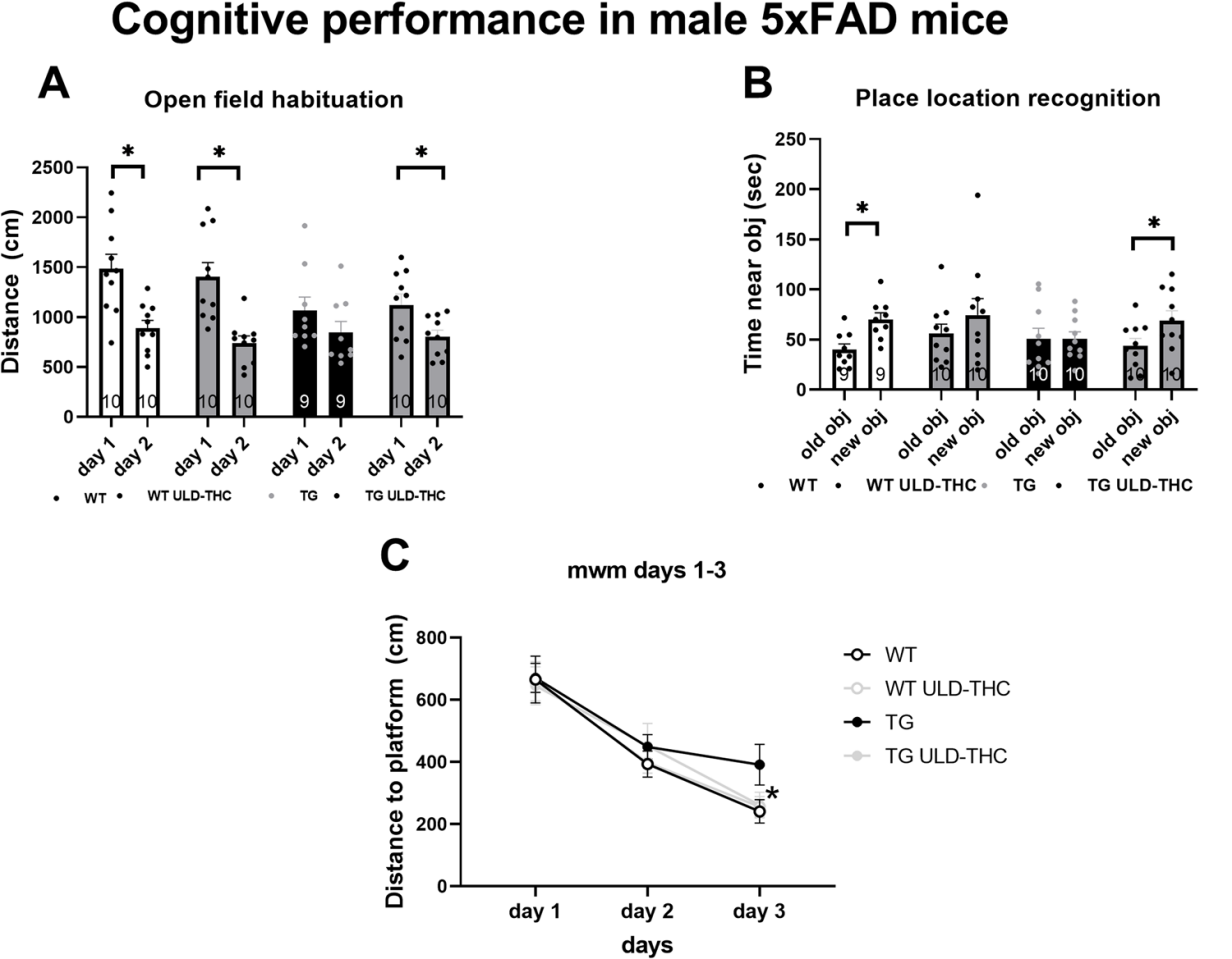
Effect of ULD-THC on cognitive performance in male 5xFAD mice. I*n the Open fied habituation*, two way anova yielded significant diferences. WT mice walked less on the second day (indicating better cognitive performance) as well as treated TG mice, while TG mice did not differ between the day (**A**). *In the novel location test*, paired t-test between the days indicated that WT mice and treated TG mice spent more time near the new object, while TG mice did not show such preference (**B**). In the MWM repeated anova showed that, TG mice took a longer path to reach the platform on day 3 compared to WT, with TG-treated mice showing some beneficial effect on the last day (**C**). Results are represented as MEAN+/-SEM. * represents *<0.05

*The Open Field Habituation test* (Fig. 1A) revealed that AD-treated showed better cognitive performance (bigger distance delta between days) than untreated AD mice. On the first day, the mice in either group moved freely in the arena, and there were no significant differences between the groups in the amount of movement in the arena (indicating no difference in motor performance. F (3, 35) = 2.363, *p* > 0.05). On the 2nd day, only vehicle-treated WT [paired two-way t-test (t = 5.589, df = 9), *p* = 0.0003], THC-treated WT [paired two-way t-test (t = 6.139, df = 9), *p* = 0.0002] and THC-treated AD [paired two-way t-test (t = 3.648, df = 9), *p* = 0.0053] mice showed a decrease in exploration, indicating intact cognitive performance. While vehicle-treated AD did not differ between the days, exhibiting reduced spatial memory retention [paired two-way t-test (t = 2.244, df = 8), *p* > 0.05]. Similarly, *in the Place recognition test* (Fig. 1B), on day 1 of the assay, all groups spent a similar time examining the objects (F (3, 36) = 0.8789, *p* > 0.05). On day 2, only vehicle-treated WT [paired two-way t-test, (t = 2.886, df = 8) *p* = 0.0203) and THC-treated AD [paired two-way t-test (t = 2.861, df = 9), *p* = 0.0188] mice spent more time exploring the object in the new location. in exploration, indicating intact cognitive performance. While vehicle-treated AD [paired two-way t-test (t = 0.004048, df = 9), *p* > 0.05] or WT [paired two-way t-test (t = 0.9259, df = 9), *p* > 0.05] did not differ, they showed a preference toward the object in the new location, exhibiting reduced spatial associative memory.

In the *Morris water maze* (Fig. 1C), which measures learning ability and spatial memory, a repeated measures ANOVA test found significant differences (F (2, 72) = 56.86, *p* < 0.0001). LSD post hoc analysis revealed that on the 3rd day, vehicle-treated AD mice had slower learning than the WT group (*p* = 0.04), with a trend toward better performance of the AD-treated mice compared to TG vehicle-treated AD (*p* = 0.08). while the AD treated did not differ from the WT group. No difference between the groups was observed on the probe day.

There was also no difference between the groups in working memory tested by the Y-maze spontaneous alternation test (F (3, 36) = 0.6332, *p* > 0.05).

### Preventive treatment with ULD-THC modifies hippocampal-neuroinflammation in male 5xFAD mice, with no effect on PFC-neuroinflammation

**Fig. 2:**
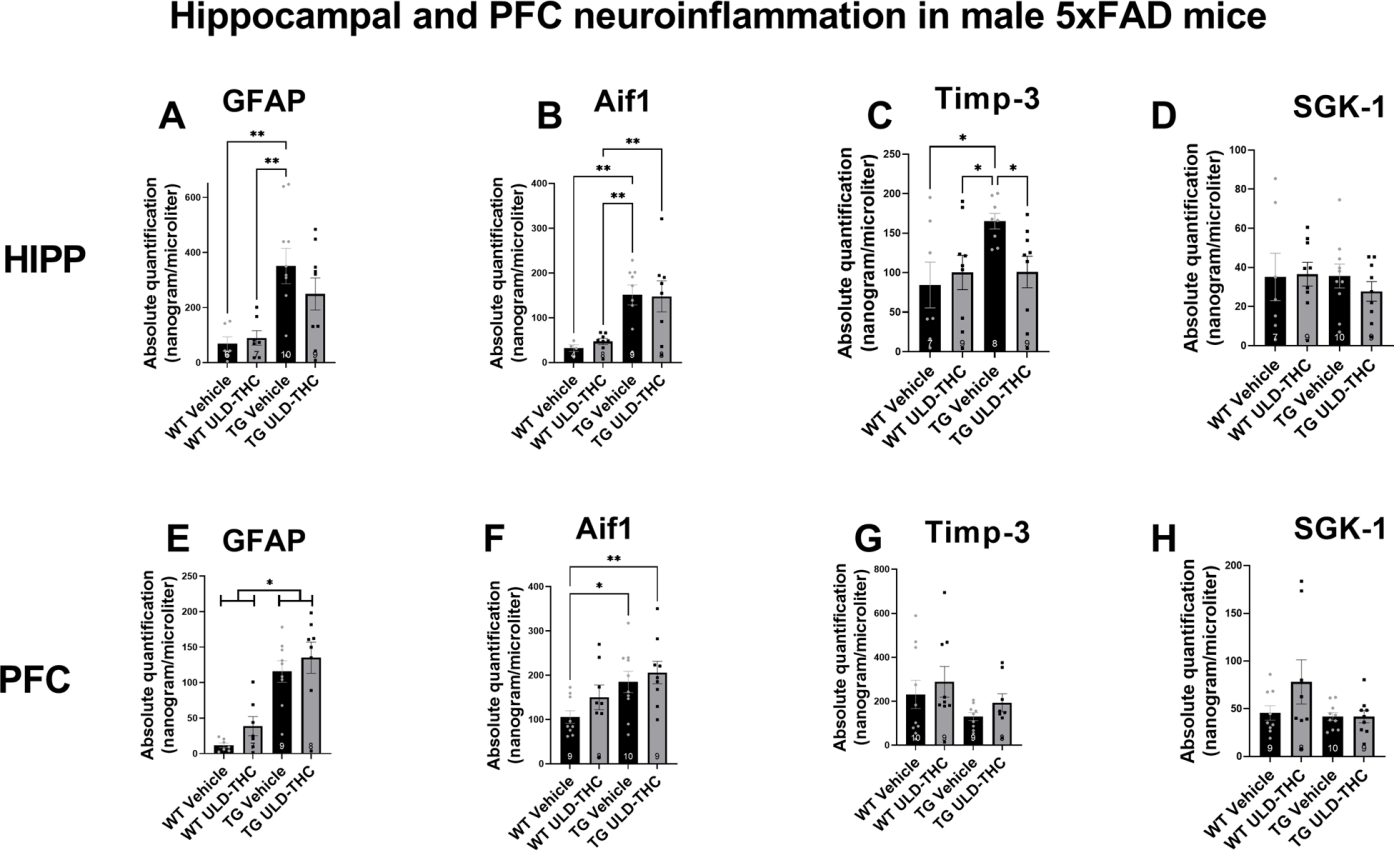
Effect of ULD-THC on neuroinflammatipn in male 5xFAD mice. *In the hippocampus (****A****-****D****)*, *in the GFAP* astrocyte marker, TG Non-treated mice had a significantly higher expresiion of GFAP compared to WT, while treated- TG mice were not significantly different from WT (A). *In the Aif1 microglial* marker, TG Non-treated mice had a significantly higher expression of GFAP compared to WT, while treated-TG mice were not significantly different from WT (B). *In the Timp-3*, TG Non-treated mice had a significantly higher expression compared to both WT and treated-TG mice (**C**). No significant difference was found in SGK-1 (**D**). *In the PFC (****E****-****F****) in the GFAP* astrocyte marker, TG Non-treated and treated mice had a significantly higher expresiion of GFAP compared to WT, (**E**). *In the AIF1 microglial* marker, TG Non-treated and treated mice had a significantly higher expresiion of GFAP compared to WT (**F**). No significant difference was found in either TIMP-3 (**G**) and SGK-1 (**H**). Results are represented as MEAN+/-SEM. * represents *<0.05. Results are represented as MEAN+/-SEM. * represents *<0.05

First, we examined glial markers – GFAP for astrocytes and AIF1 for microglia. In the hippocampus, in astrocytes (Fig. 2A), we saw a significant difference between the groups [F (3, 28) = 6.107, *P* = 0.0025], with LSD post-hoc showing that non-treated AD mice exhibiting highly elevated expression compared to vehicle-treated WT (*p* = 0.0070) and THC-treated WT (*p* = 0.0093). Importantly, THC-treated AD mice were not significantly different from WT (*p* = 0.13). when looking at microglia (Fig. 2B), as expected, there was also a significant difference between the groups [F (3, 27) = 3.916, *P* = 0.0192], with LSD post-hoc showing non-treated AD and THC-treated AD mice exhibiting elevated expression compared to vehicle-treated WT (*p* = 0.0383 and *p* = 0.0502, respectively) and compared to THC-treated WT (*p* = 0.03 for both).

In the PFC, in astrocytes (Fig. 2E), we saw a significant difference between the groups [F (3, 27) = 13.93, *P* < 0.0001]. However, in contrast to the hippocampus, where ULD-THC treatment mice did not differ from WT, in the PFC, LSD post-hoc showed both non-treated AD mice (*P* < 0.0001) and treated-AD mice (*P* < 0.0001) exhibited highly elevated expression compared to vehicle-treated WT. When looking at microglia (Fig. 2F), the same trend appeared. There was also a significant difference between the groups [F (3, 32) = 3.544, *P* = 0.0253], with LSD post-hoc showing both non-treated AD (*p* = 0.0196) and THC-treated AD mice (*p* = 0.0048) exhibiting elevated expression compared to vehicle-treated WT.

Next, we examined tissue inhibitor of metalloproteinase 3 (TIMP3) (Fig. 2C). TIMP-3 plays a complex role in AD and aging. On the one hand, it inhibits Matrix metalloproteinases (MMPs)-induced inflammation, has a neuroprotective effect [31] and is associated with protection against neuroinflammation [32]. On the other hand, it inhibits APP cleavage, resulting in increased levels of Aβ [42]. Our results in the male hippocampus show a significant difference between the groups [F (3, 29) = 2.83, *P* = 0.05]. Non-treated AD mice exhibited highly elevated expression levels of TIMP-3 compared to vehicle-treated WT (*p* = 0.0140) and THC-treated WT (*p* = 0.0.0330). Importantly, THC-treated AD mice had lower expression compared to non-treated AD mice (*p* = 0.0347). There was no significant treatment effect on SGK expression (F (3, 31) = 0.3302, *p* > 0.05; Fig. 2D). There was no significant difference between the groups in the PFC of either TIMP-3 (F (3, 32) = 1.510, *p* > 0.05; Fig. 2G) and SGK (F (3, 32) = 2.129, *p* > 0.05; Fig. 2H)

### Preventive treatment with ULD-THC improves Spatial hippocampal-dependent memory and PFC-dependent working memory in female 5xFAD mice

**Fig. 3:**
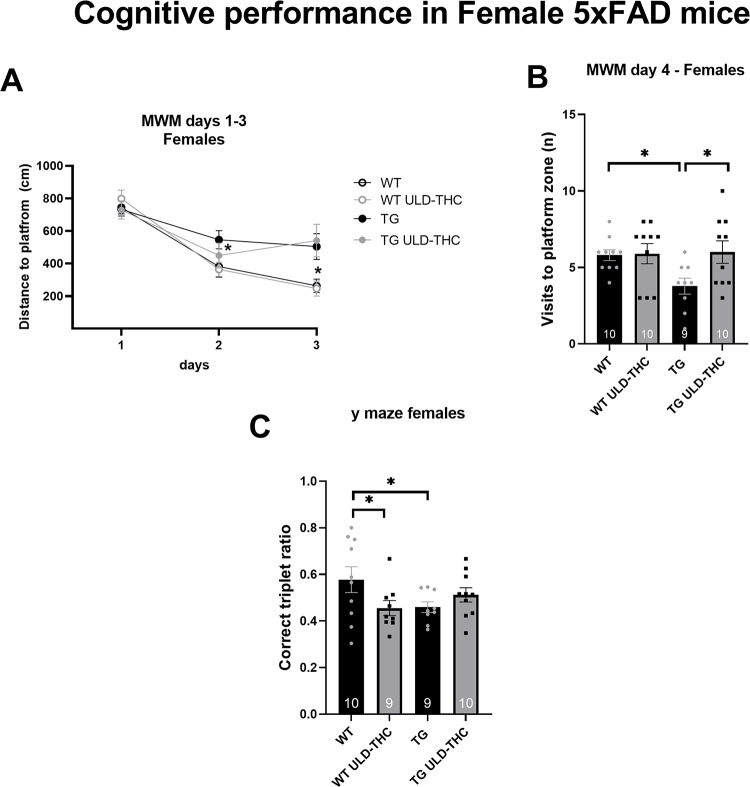
Effect of ULD-THC in females. In the MWM repeated anova showed that, TG mice took a longer path to reach the platform compared to WT on days 2& 3. Treated TG mice did not differ from WT on day 2, which may indicate a slight improvemnt in their performance compared to non-treated TG mice. (**A**). On the ‘probe’ test, TG mice visited the platform zone less both compared to WT and treated-TG mice *In the Y-maze test. one way* anova showed that AD mice had less correct triplets ratio, indicating worse cognitive performance, compared to WT mice. Treated TG mice were not different compared to WT mice (**C**). Results are represented as MEAN+/-SEM. * represents *<0.05

In the *Morris water maze* (Fig. 3A), which measures learning ability and spatial memory, a repeated measures ANOVA test found significant differences (F (2, 18) = 7.517, *p* = 0.0042). LSD post hoc analysis revealed that on the 2nd day, vehicle-treated AD mice had slower learning than the WT group (*p* = 0.05), while the AD treated mice did not differ from the WT group. On the 3rd day, both AD groups were significantly different compared to the WT groups. In the *prob trial* on the 4th day (Fig. 3B), there was a significant effect for the treatment [F (3, 35) = 3.10, *p* = 0.0391]. LSD post hoc analysis revealed that non-treated AD mice visited significantly fewer times in the platform zone than vehicle-treated WT (*p* = 0.0220) and THC-treated WT (*p* = 0.0.0167). Importantly, THC-treated AD mice had spent more time in the correct location compared to non-treated AD mice (*p* = 0.0125).

In *the Y-maze*, spontaneous alternation, testing for working memory, planned contrast revealed significant differences between the WT group and the vehicle-treated AD mice (*p* = 0.03), with no difference between WT and TG-treated AD mice (*p* = 0.22).

There was also no effect for treatment in the open field habituation test (paired two way t-test between day: *p* > 0.05 for the treatment group) and the Place recognition test (paired two way t-test between days: *p* > 0.05 for treatment group).

### Preventive treatment with ULD-THC modifies hippocampal and PFC-neuroinflammation in female 5xFAD mice

First, we examined glial markers – GFAP for astrocytes and AIF1 for microglia.

**Fig. 4:**
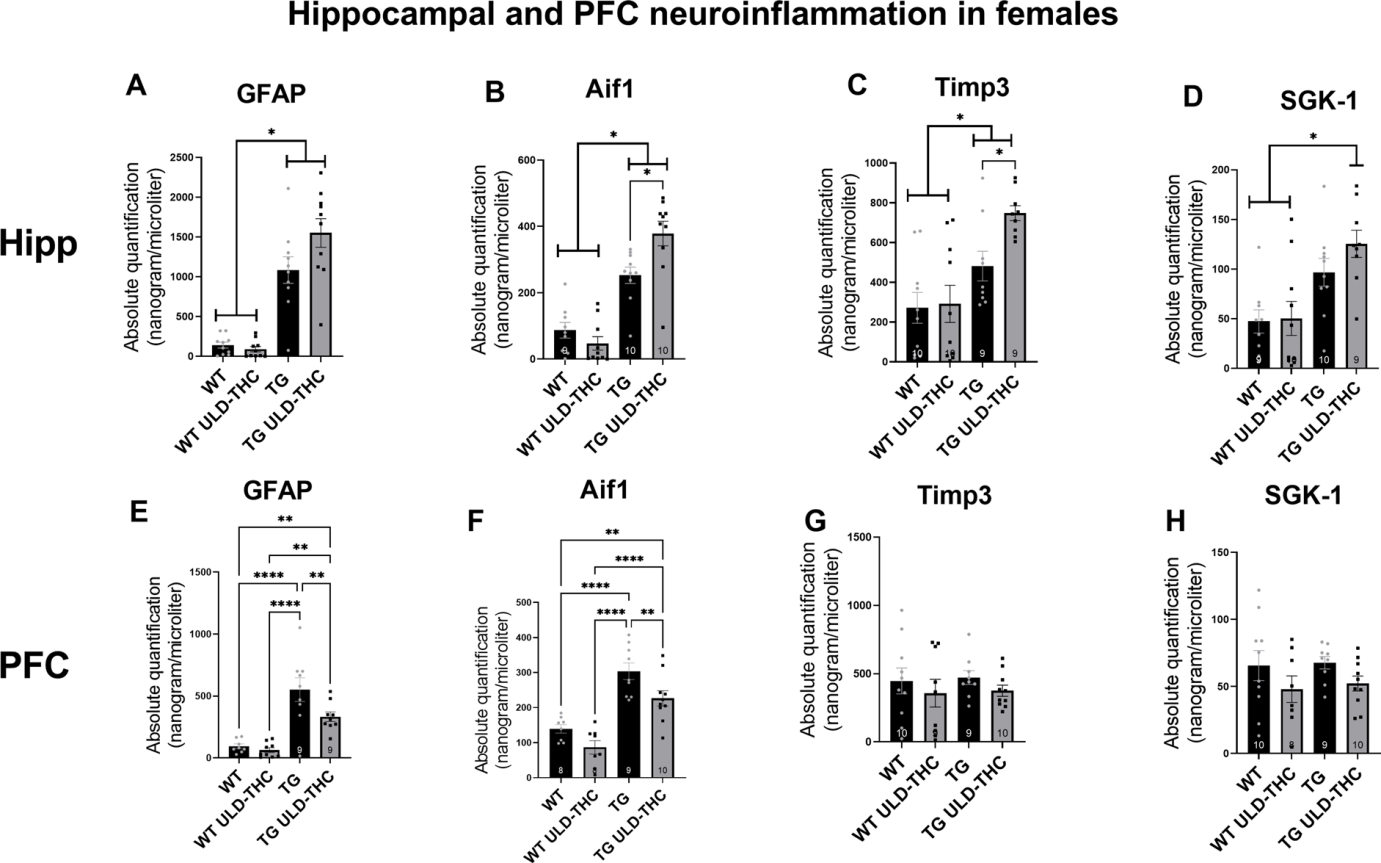
Effect of ULD-THC on hippocampal neuroinflammatipn in female 5xFAD mice.*In the GFAP* astrocyte marker, in the hippocampus (**A**) both treated and non-treated TG mice had higher expresiion of GFAP compared to WT, while in the PFC (**E**) TG Non-treated mice had a significantly higher expresiion of GFAP compared to WT and treated TG mice. I*n the AIF1* microglial marker, in the hippocampus (**B**) THC-treated TG mice had higher expression compared to all other groups. While in the PFC, TG Non-treated mice had a significantly higher expresiion of *AIF1* compared to WT and treated TG mice(**F**). In hippocampal TIMP3 (**C**) THC-treated TG mice had higher expression compared to all other groups, and there was no difference in the PFC (**G**). In the SGK1 in the hippocampus (**D**) both treated and non-treated TG mice higher expression of SGK1 compared to WT, and there was no difference in the PFC (H). Results are represented as MEAN+/-SEM. * represents *<0.05

In hippocampal astrocytes (Fig. 4A), we saw a significant difference between the groups [F (3, 36) = 32.98, < 0.0001], with both non-treated AD mice and treated-AD mice exhibiting elevated expression compared to vehicle-treated WT and THC-treated WT (< 0.0001 for all comparisons).

In the PFC (Fig. 4E), one-way ANOVA also yielded a significant difference between the groups [F (3, 31) = 17.53, *p* < 0.0001]. Interestingly, post-hoc analysis revealed that while non-treated AD still has significantly higher expression of GFAP compared to vehicle-treated WT and THC-treated WT (< 0.0001 for both), there was a down-regulation of GFAP in the treated AD mice compared to non-treated AD-mice (*p* = 0.0074). Similar results were obtained in the marker of microglia, AIF1. In the Hippocampus (Fig. 4B), we saw a significant difference between the groups [F (3, 35) = 31.67, *p* < 0.0001], with both non-treated AD mice and treated-AD mice exhibiting elevated expression compared to vehicle-treated WT and THC-treated WT (< 0.0001 for all comparisons) and treated-TG mice had higher AIF1 expression compared to non-treated TG mice (*p* = 0.0117). As with GFAP, the treatment had the opposite effect in the PFC (Fig. 4F). One-way ANOVA yielded a significant difference between the groups [F (3, 31) = 21.50, *p* < 0.0001], and post-hoc analysis revealed that while non-treated AD still has significantly higher expression of GFAP compared to vehicle-treated WT and THC-treated WT (< 0.0001 for both), there was a down-regulation of GFAP in the treated AD mice compared to non-treated AD-mice (*p* = 0.0091). Combined, these results suggest an anti-inflammatory effect of ULD-THC in females only in the PFC, with no effect or even greater gliosis in the hippocampus after treatment in females.

In order to identify possible sex differences, we also performed a three-way ANOVA for GFAP for astrocytes and AIF1 for microglia (see supplementary data). In hippocampal astrocytes (Figure S1A), we saw a significant treatment*sex interaction [F(3, 64) = 30.67, < 0.0001]. LSD post-hoc analysis revealed that both male and female non-treated AD mice exhibited elevated expression compared to vehicle-treated WT mice, with female treated-AD mice exhibiting the highest level of GFAP level.

In the PFC (Figure S1E), we also saw a significant treatment*sex interaction [F(3, 58) = 9.443, < 0.0001]. LSD post-hoc analysis showed that both male and female non-treated AD mice exhibiting elevated expression compared to vehicle-treated WT, with female treated and non-treated-AD mice exhibited higher GFAP expression levels compared to male treated and non-treated-AD mice. Interestingly, female treated-AD mice exhibiting the downregulation of GFAP levels compared to non-treated AD-mice. Similar results were obtained in the marker of microglia, AIF1. In the Hippocampus (figure S1B) we saw a significant treatment*sex interaction [F(3, 63) = 5.650, *p* < 0.0017]. LSD post-hoc analysis showed that both male and female non-treated AD mice exhibiting elevated expression compared to vehicle-treated WT with female treated and non-treated-AD mice exhibited higher AIF1 expression levels compared to male treated and non-treated-AD mice and female treated-AD mice exhibiting the upregulation of AIF1 levels compared to non-treated AD-mice. As with GFAP, the treatment had the opposite effect in the PFC (figure S1F). Three-way ANOVA yielded a significant treatment*sex interaction [F (3, 60) = 6.949, *p* < 0.0004]. LSD post-hoc analysis showed that both male and female non-treated AD mice exhibiting elevated expression compared to vehicle-treated. Importantly, only female treated-AD mice exhibiting a downregulation of AIF1 levels compared to non-treated AD-mice. Combined, these results suggest an anti-inflammatory effect of ULD-THC in females only in the PFC, with no effect or even greater gliosis in the hippocampus after treatment in females.

Our result coincides with other studies showing site-specific differences between the sexes in this mode. In the PFC of 7-month-old 5xFAD mice, the upregulation of allograft inflammatory factor 1 (*Aif1*), a marker of microglial activation, was slightly higher in females than males. A significantly higher upregulation of glial fibrillary acidic protein (*Gfap*), an astrocyte marker, was found in females compared to males [29].

Next, we examined tissue inhibitor of metalloproteinase 3 (TIMP3). As mentioned before, TIMP-3 can be upregulated in inflammatory states as a compensatory mechanism, promoting neuroinflammation [32] but its upregulation can also increase Aβ levels [42]. Our results in the hippocampus (Fig. 4C) show a significant difference between the groups [F (3, 34) = 8.488, *p* = 0.0002]. WT mice exhibited lower expression levels of TIMP-3 compared to non-treated TG (*p* = 0.005) and THC-treated TG (*p* < 0.0001), and THC-treated AD mice had higher expression compared to non-treated AD mice (*p* = 0.0205). There was no significant difference between the groups in the PFC (Fig. 4G).

Lastly, we examined SGK-1 expression, which is known to regulate microglia and facilitate neuroprotection [43, 44]. In the Hippocampus (Fig. 4C), one-way ANOVA yielded a significant difference between the groups. There was no significant difference between the groups in the PFC (Fig. 4H).

In order to identify possible sex differences, we also performed a three-way ANOVA on TIMP3 and SGK-1. In hippocampal TIMP3 (Figure S1C) there was a significant sex*group interaction [F(3, 65) = 6.858, *p* = 0.0004]. LSD post-hoc analysis showed that all males had lower expression of TIMP3 compared to females. In females, WT mice exhibited lower expression levels of TIMP-3 compared to non-treated TG and THC-treated TG, and THC-treated AD mice had higher expression compared to non-treated AD mice. There was no significant difference between the males in different treatment groups. In the PFC (Figure S1G), there was also a significant sex effect [F(1, 66) = 18.17,*P* < 0.0001], with female mice exhibiting higher expression of TIMP3 compared to male mice (*p* < 0.05). In hippocampal SGK-1 expression, (Figure S1D), Three-way ANOVA yielded a significant treatment*sex interaction [F(3, 65) = 6.038, *p* < 0.00011]. Post hoc analysis showed that female AD mice, both treated and un-treated, had higher expression of SGK-1 compared to WT female mice and to all male mice, both WT and AD. Interestingly, in females only, there was a trend toward an upregulation on SGK-1 in treated-AD mice compared to non-treated mice. In males, there was no significant difference between the groups. There was no significant difference between the any of the group or sexes in the PFC (Figure S1H).

**Fig. 5:**
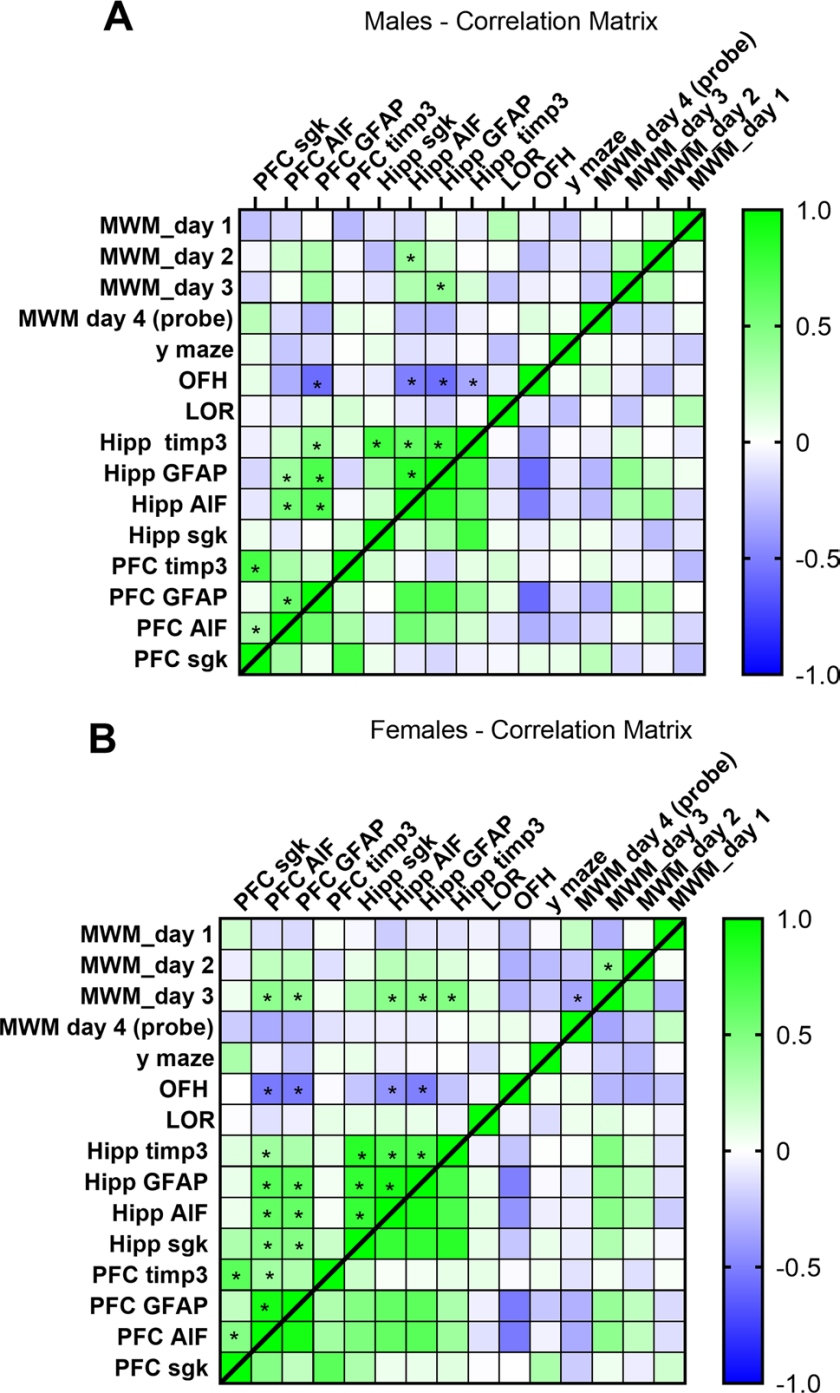
Correlation matrices for males (**A**) and females (**B**) showing Pearson correlations between behavioral measures and molecular markers in the hippocampus (Hipp) and prefrontal cortex (PFC). Correlation coefficients (r) range from –1 to +1 and are visualized using a color scale (green = positive correlation, blue = negative correlation). Significant correlations are indicated by an asterisk (*p < 0.05, two-tailed). Sample sizes ranged from 30–40 animals per sex, with variation due to missing values

The relationship between behavioral and molecular variables was analyzed using Pearson’s correlation coefficient (two-tailed, *p* < 0.05). Separate correlation matrices were generated for male (Fig. 5A) and female (Fig. 5B) mice to assess sex-specific patterns. Across both sexes, hippocampal markers (GFAP, AIF, sgk, and timp3) showed significant intercorrelations, reflecting consistent within-region molecular associations. In addition, in both males and females, performance in the open field habituation (OFH) task correlated positively with hippocampal indices (*p* < 0.05).

However, distinct sex-specific differences were observed. In males (Figure A), significant correlations were largely restricted to hippocampal markers and their association with OFH performance, suggesting a primarily hippocampal network of molecular–behavioral relationships. In females (Figure B), the pattern extended beyond the hippocampus: multiple prefrontal cortex (PFC) markers correlated significantly with hippocampal indices as well as with behavioral outcomes, including Morris water maze (MWM) performance and OFH (*p* < 0.05). This broader network highlights a more integrated prefrontal–hippocampal contribution to cognitive and exploratory behavior in females, compared to the hippocampus-centered profile observed in males.

## Discussion

In this study, we sought to evaluate the sex-dependent effect of a sub-chronic preventive treatment of ULD-THC in an AD mouse model. 3 injections were administered once a month from 3 to 5 months of age, and behavioral and molecular analyses were conducted at 6 months.

Our behavioral assessments included tests for spatial memory, working memory, and contextual learning, providing a comprehensive overview of cognitive domains affected by the treatment. We show that both male and female mice had behavioral improvement in the Morris Water Maze (MWM). However, Sex-specific improvements were observed as male 5xFAD-treated mice demonstrated improvement in several hippocampus-dependent tasks, such as the Place recognition test and the Open field habituation, while females demonstrated improvement in the short-term memory Y-maze. Interestingly, in females, THC has shown a dual effect, showing improvement in the AD mice model, while resulting in diminished capacities in the WT mice. This dual effect has been demonstrated previously in old vs. young mice ([16] as well as in the AD model when the treatment is given after the disease progress [17].

In accordance with our results, sex differences in AD and its preclinical models are well-documented, with distinct patterns observed in both behavioral performance and underlying neural mechanisms [45]. A recent study in humans showed a sex-dependent difference between men and women in their performance in immediate and delayed recall memory tests, which correlates to damage in the PFC [46]. This was also shown in animal models. For example, in the 5xFAD animal model, in the Morris water maze, which usually represents hippocampus-dependent spatial learning, analysis of female 5xFAD mice search strategies in the probe trial yielded significant sex differences, with female mice exhibiting more non-hippocampal-dependent (egocentric) strategies compared to males [47].

The hippocampus and prefrontal cortex (PFC) are two critical brain regions implicated in short—and long-term spatial and working memory, and their roles may differ based on sex and cognitive demands. Thus, after finalizing the behavioral examination, we explored the relationship between sex-specific behavioral outcomes and molecular markers. To investigate the underlying molecular mechanisms, we analyzed the expression of neuroinflammation-related genes that play critical roles in AD. Indeed, we observed sex-dependent site-specific effects of the treatment on neuroinflammation that corresponded with the observed cognitive improvement.

First, we saw a sex-specific effect of glial cells’ response to the ULD-THC in the hippocampus of male and female mice. In males, we observe a reduction in glial cells, specifically astrocytes, in the hippocampus, with males showing downregulation in the expression of hippocampal glial cells and no change (and even a slight upregulation) of hippocampal glial cell expression in females.

Glial cells are known to demonstrate marked sex differences from early development to adulthood [48, 49] and in reaction to an assault [50]. This differential activation is related to cognitive functioning [51]. A recent study showed hippocampal astrocyte activation has opposite effects on cognitive function in male and female mice. A reduction in hippocampal astrocyte activity impaired memory in females but enhanced memory in males, while increasing hippocampal astrocyte activity enhanced memory in females in the MWM, specifically in the probe trial [52]. This study can provide an explanation for the opposite effect we saw in astrocyte expression in the hippocampus of ULD-THC-treated male and female 5xFAD mice (lower in males and higher in females), while both male and female-treated mice showed an improvement in hippocampus-dependent learning in the MWM.

The reduced glial cell expression in our 5xFAD THC-treated males may indicate a decrease in neuroinflammation, reducing the need for anti-inflammatory genes such as SGK1. Additionally, a reduction in TIMP3 is evident, which aligns with the reduced inflammation and suggests lower amyloid levels. TIMP-3, which inhibits Matrix metalloproteinases (MMPs)-induced inflammation, has a neuroprotective effect [31] and is associated with protection against neuroinflammation [32]. However, its effect in AD is less clear, as it is upregulated in AD human patients and AD mouse models and is known to inhibit APP cleavage, resulting in increased levels of Aβ [42]. On the other hand, no decrease in inflammation is observed in this region in females; on the contrary, glial activation appears to rise. As a compensatory response, there is an increase in markers associated with combating inflammation, such as TIMP3. Interestingly, a significant rise in SGK1 is also observed in this region in treated females, which is absent in males. SGK1 also plays a part in regulating microglia and has been known to facilitate neuronal function [53] and has a neuroprotective role [43, 44]. Thus, SGK1 upregulation in treated mice can be a treatment-induced compensatory mechanism to the higher inflammatory state in the hippocampus.

Notably, as opposed to the hippocampus, the PFC-treated female mice exhibited reduced inflammation, as evidenced by the downregulation of glial markers. This reduction in inflammation was not evident in male mice. Consequently, in females, there is no significant change in anti-inflammatory gene expression in this region, as inflammation has already subsided.

The correlation analyses further reflect the distinct patterns across sexes and brain regions observed in the behavioral and molecular examination. In males, behavioral improvements and molecular changes were largely confined to the hippocampus, consistent with correlations linking hippocampal markers to hippocampus-dependent tasks. In females, correlations revealed a broader network involving both hippocampal and PFC markers, reflecting the observed improvements in both hippocampal and PFC-related tasks, as well as the molecular changes in these regions. However, it should be noted that the interpretation of these correlations is limited by the inherent variability of behavioral paradigms and the complexity of neuroinflammatory processes and thus should be considered with caution. In sum, the correlations highlight sex- and region-specific associations between molecular markers and behavioral performance.

Our research demonstrates ULD-THC’s unique potential as an efficient method of treating Alzheimer’s disease. While conventional therapies primarily address symptoms after significant disease progression, our findings suggest that treatment with an ultra-low dose of THC at an early stage may have neuroprotective effects, potentially reducing the onset and progression of the disease. Crucially, ULD-THC is not the same as conventional high-dose THC treatments, which are more dangerous and have more adverse effects, especially in older people [20, 54]. Utilizing an ultra-low dosage, we hope to maximize THC’s positive benefits while reducing its negative ones. These results are in line with the effects seen in old female mice treated with ULD-THC [16, 32]. We have previously shown that a single ULD-THC treatment in old mice helps alleviate age-related cognitive decline [16]. This cognitive improvement went hand-in-hand with an upregulation of SGK-1 and TIMP-3 in the hippocampus of ULD-THC-treated old female mice [32].

**Fig. 6:**
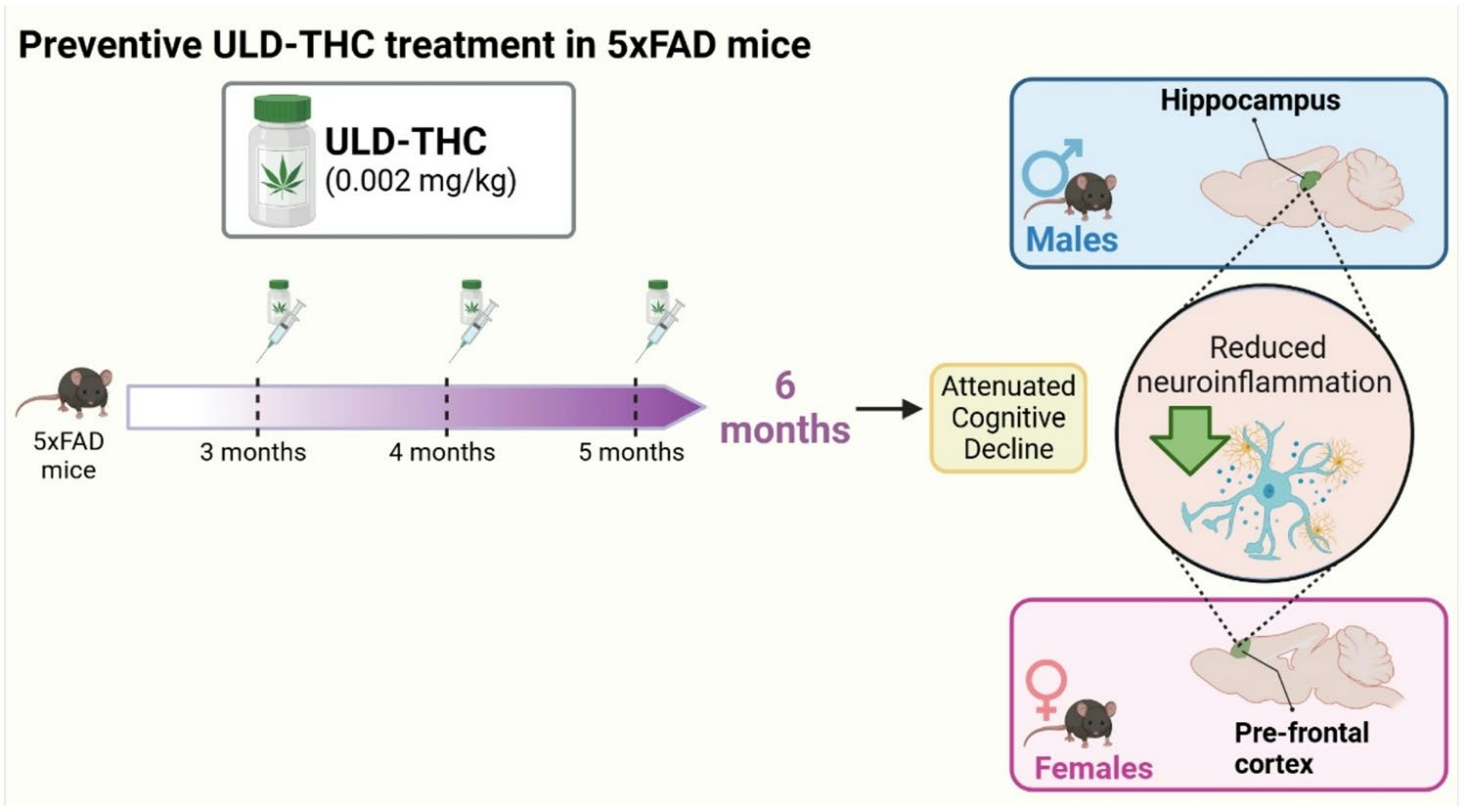
Schematic representation of sex-dependent effects of preventive ULD-THC treatment. 5xFAD mice begin developing amyloid pathology at 2–3 months, with cognitive deficits emerging by 6 months. To evaluate preventive effects, ULD-THC was administered monthly from 3 to 5 months of age. At 6 months, treatment attenuated AD-related cognitive decline in both sexes, accompanied by sex- and region-specific reductions in neuroinflammation. Males exhibited anti-inflammatory effects in the hippocampus, while females showed reduced inflammation in the PFC. Image created with BioRender.com

To conclude, our results demonstrate a distinct site-dependent effect of ULD-THC treatment in males and females, with females showing reduced inflammation markers in the PFC while males showing a noticeable effect in the Hippocampus (Fig. 6). These changes might be related to the different cognitive effects that we saw in the behavioral tests between the sexes.

Collectively, our results point towards a unique sex-dependent pattern of neuroinflammation after preventive treatment with ULD-THC. It serves as a step toward understanding sex-specific responses to preventive treatments in AD, providing novel insights while laying the groundwork for future, more comprehensive analyses.

## Supplementary Information

Supplementary Material 1.

## Data Availability

The datasets used and/or analysed during the current study are available from the corresponding author on reasonable request.

## Abbreviations

AD: Alzheimer’s disease.
ULD-THC: Ultra-low-dose of tetrahydrocannabinol.
PFC: Prefrontal cortex.
Aβ: Beta-amyloid.
SGK1: Serum-and glucocorticoid-inducible kinase 1.
TIMP: 3-Tissue Inhibitor of Metalloproteinases 3.
MWM: Morris Water Maze.
YM: Y maze.
PLT: Place recognition test.
OFH: Open-field habituation.
