# Supplementary material for: Sex-dependent effects of ultra-low-dose-THC preventive treatment on neuroinflammation and cognitive decline in 5xFAD mice
Source: Biol Sex Differ. 2026 Jan 3;17:20. doi: 10.1186/s13293-025-00815-3 (PMC12866039; doi:10.1186/s13293-025-00815-3)

Preventive treatment with ULD-THC modifies hippocampal and PFC-neuroinflammation in male and female 5xFAD mice

In order to identify possible sex differences, we performed a three-way Anova on all the genes.

First, we examined glial marker – GFAP for astrocytes and AIF1 for microglia.

In hippocampal astrocytes (Figure S1A), we saw a significant difference between the treatment groups [F(3, 36) = 32.98, <0.0001], between sex [F(1, 64) = 48.76, <0.0001], as well as Treatment x Sex interaction [F(3, 64) = 30.67, <0.0001]. LSD post-hoc analysis showed that both male and female non-treated AD mice exhibiting elevated expression compared to vehicle-treated WT (p=0.08 for males and p<0.0001 for females). Female treated and non-treated-AD mice exhibited higher GFAP expression levels compared to male treated and non-treated-AD mice (p=<0.0001), with female treated-AD mice exhibiting the highest level of GFAP level (p=<0.0001).

In the PFC (Figure S1E), we saw a significant difference between the treatment groups [F(3, 58) = 21.81, <0.0001], between sex [F(1, 58) = 37.20 <0.0001], as well as Treatment x Sex interaction [F(3, 58) = 9.443, <0.0001]. LSD post-hoc analysis showed that both male and female non-treated AD mice exhibiting elevated expression compared to vehicle-treated WT (p=0.09 for males and p<0.0001 for females). Female treated and non-treated-AD mice exhibited higher GFAP expression levels compared to male treated and non-treated-AD mice (p=<0.0001). Interestingly, female treated-AD mice exhibiting the downregulation of GFAP levels compared to non-treated AD-mice (p=0.0004).

Similar results were obtained in the marker of microglia, AIF1. In the Hippocampus (figure S1B), we saw a significant difference between the treatment groups [F(3, 63) = 17.05, p<0.0001], as well as Treatment x Sex interaction [F(3, 63) = 5.650, p<0.0017]. LSD post-hoc analysis showed that both male and female non-treated AD mice exhibiting elevated expression compared to vehicle-treated WT (p=0.01 for males and p<0.0001 for females). Female treated and non-treated-AD mice exhibited higher AIF1 expression levels compared to male treated and non-treated-AD mice (p=<0.0001). Furthermore, female treated-AD mice exhibiting the upregulation of AIF1 levels compared to non-treated AD-mice (p=0.0006). As with GFAP, the treatment had the opposite effect in the PFC (figure S1F). Three-way ANOVA yielded a significant difference between the groups [F(3, 60) = 30.74, p<0.0001], between sex [F (1, 60) = 24.36, p<0.0001], as well as Treatment x Sex interaction [F (3, 60) = 6.949, p<0.0004]. LSD post-hoc analysis showed that both male and female non-treated AD mice exhibiting elevated expression compared to vehicle-treated WT (p=0.01 for males and p<0.0001 for females). Importantly, only female treated-AD mice exhibiting a downregulation of AIF1 levels compared to non-treated AD-mice (p=0.01). Combined, these results suggest an anti-inflammatory effect of ULD-THC in females only in the PFC, with no effect or even greater gliosis in the hippocampus after treatment in females.

Next, we examined a tissue inhibitor of metalloproteinase 3 (TIMP3). Our results in the hippocampus (Figure S1C) show a significant difference between the groups [F(3, 65) = 7.640, p=0.0002], sex [F(1, 65) = 70.23, p=0.0001], and Treatment x Sex interaction [F(3, 65) = 6.858, p=0.0004]. LSD post-hoc analysis showed that all males had lower expression of TIMP3 compared to females (p>0.03). In females, WT mice exhibited lower expression levels of TIMP-3 compared to non-treated TG (p =0.01) and THC-treated TG (p<0.0001), and THC-treated AD mice had higher expression compared to non-treated AD mice (p=0.001). There was no significant difference between the males in different treatment groups.

In the PFC (Figure S1G), there was a significant sex effect [F(1, 66) = 18.17,P<0.0001], with female mice exhibiting higher expression of TIMP3 compared to male mice (p<0.05). There was no significant difference between the treatment groups.

Lastly, we examined SGK-1 expression. In the Hippocampus (Figure S1D), Three-way ANOVA yielded a significant difference between the groups [F(3, 65) = 4.276, p<0.008], between sex [F(1, 65) = 30.81, p<0.0001], as well as Treatment x Sex interaction [F(3, 65) = 6.038, p<0.00011]. Post hoc analysis showed that female AD mice, both treated and un-treated, had higher expression of SGK-1 compared to WT female mice (p<0.001) and to all male mice, both WT and AD (p<0.0008). Interestingly, In females only, there was a trend toward an upregulation on SGK-1 in treated-AD mice compared to non-treated mice (p=0.08). In males, there was no significant difference between the groups. There was no significant difference between the any of the group or sexes in the PFC (Figure S1H).

**Body weight**

A three-way Anova was performed in order to detect changes in body weight (Figure S2). As expected, there was a significant main effect for sex [f(4.347, 312.968) = 26.11, p<0.01], with males exhibiting higher weights compared to females. There was also an expected main effect for group [f(13.040, 312.968)=2.715, p<0.01]. LSD Post hoc analysis indicated that both WT groups had a higher weight compared to both AD mice (p<0.01). There was no significant difference between AD-treated mice and AD-non treated mice.

**Cognitive performance**

The performance of male and Female mice was also examined together to indicate any sex-driven differences.

In the Open Field Habituation (Figure S3A) there was a significant difference between the groups [F(7, 71) = 6.465, p <0.0001]. In males, only WT, WT-treated, and TG-treated mice showed a significant difference between days, indicating better cognitive performance compared to the non-treated TG. In Females, only the WT groups had a significant difference between the days, indicating better cognitive performance of healthy vs. AD mice, regardless of treatment. There was also a significant difference between male and female AD mice, with male AD mice showing reduced cognitive performance compared to female AD mice, regardless of treatment. In the Y-maze test (Figure S3B), a significant Treatment x Sex interaction was also observed [F(1, 70) = 4.738, p < 0.03]. In females, a significant difference was observed between the WT group and the vehicle-treated AD mice, with no difference noted between the WT and TG-treated AD mice. Furthermore, WT female mice had a significantly higher correct triplet ratio compared to male WT mice.

In the Morris water maze (MWM) over the first 3 days (Figure S3C), a significant difference was observed between the groups [F(7, 72) = 2.896, p = 0.01]. In females, there was a significant difference on the 2^nd^ day, with the non-treated AD mice taking the longest to reach the platform. The overall performance of female TG mice was worse compared to that of WT female mice and male TG mice, regardless of treatment. On the 4^th^ day of the MWM (Figure S3D), a significant interaction between Treatment x Sex was observed [F(1, 68) = 5.200, p < 0.02]. In females. Non-treated TG mice showed lower cognitive performance compared to treated-AD mice. Furthermore, male WT mice had lower cognitive performance compared to male treated-WT mice and compared to female WT mice. Lastly, in the Place location recognition task (Figure S3F), there was a significant difference in the preference between the old and the new object [F(1, 71) = 5.821, p =0.01]. In males, only WT and TG-treated mice showed a preference to the new object, indicating better cognitive performance compared to the non-treated TG. There was no difference between the groups in females.

There was no preferences show to either object location [F(1, 138) = 2.652, p>0.05] on the first day of the Place location recognition task (Figure S3E),

**Place location recognition arena and objects**

A picture of the arena setting and location of the objects during the familiarization and test session


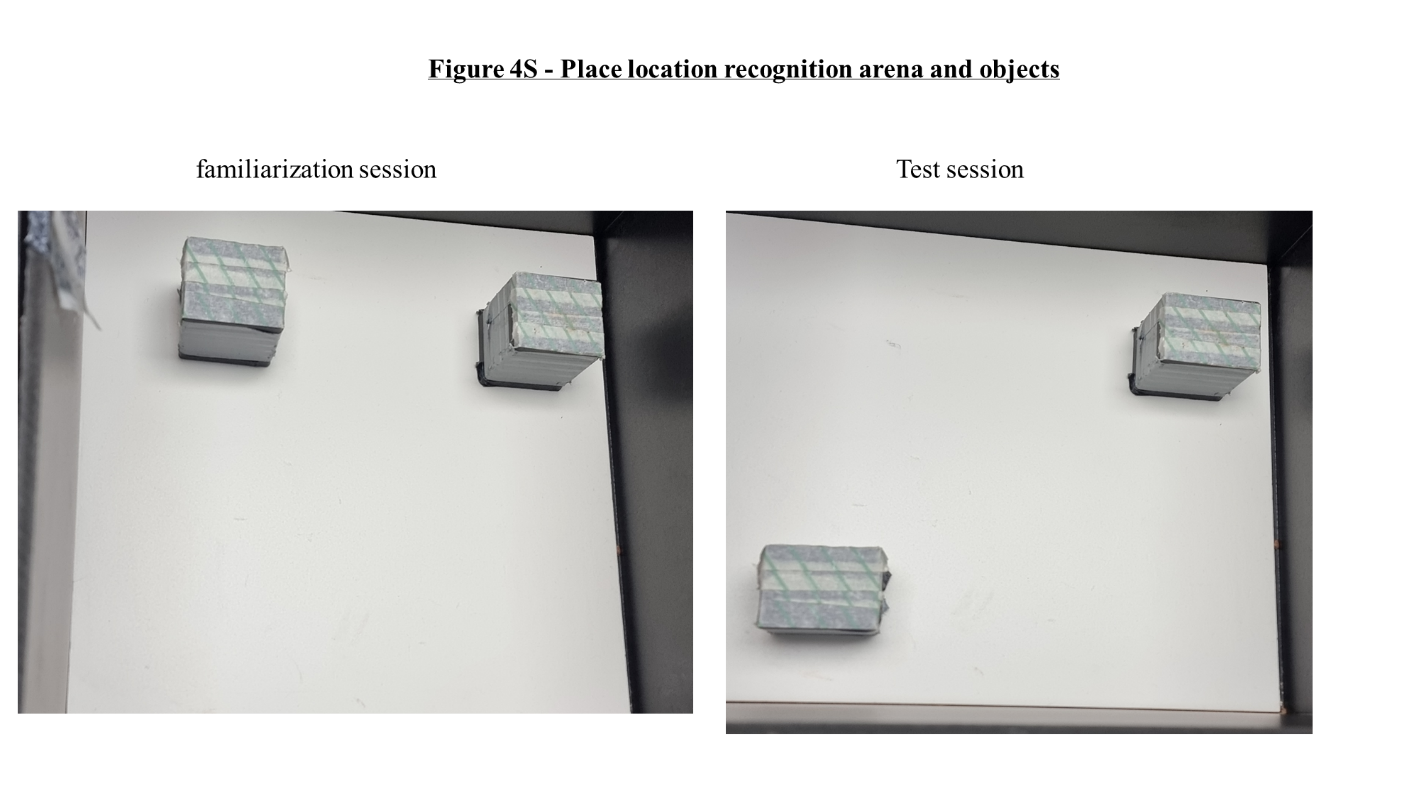

Supplement: Supplementary file 1 — Supplementary Material 1. [file 13293_2025_815_MOESM1_ESM.docx]
